# Advances in gut–brain organ chips

**DOI:** 10.1111/cpr.13724

**Published:** 2024-07-31

**Authors:** Yu Zhang, Si‐Ming Lu, Jian‐Jian Zhuang, Li‐Guo Liang

**Affiliations:** ^1^ Key Laboratory of Clinical Cancer Pharmacology and Toxicology Research of Zhejiang Province, Affiliated Hangzhou First People's Hospital, School of Medicine Westlake University Hangzhou China; ^2^ Department of Pharmacy, Affiliated Hangzhou First People's Hospital, School of Medicine Westlake University Hangzhou China; ^3^ Department of Laboratory Medicine, The First Affiliated Hospital Zhejiang University School of Medicine Hangzhou China; ^4^ Zhejiang Key Laboratory of Clinical In Vitro Diagnostic Techniques Hangzhou China; ^5^ Institute of Laboratory Medicine Zhejiang University Hangzhou China; ^6^ Centre for Clinical Laboratory The First Affiliated Hospital of Zhejiang Chinese Medical University Hangzhou China; ^7^ State Key Laboratory for Diagnosis and Treatment of Infectious Diseases, The First Affiliated Hospital Zhejiang University School of Medicine Hangzhou China; ^8^ National Clinical Research Center for Infectious Diseases, The First Affiliated Hospital Zhejiang University School of Medicine Hangzhou China

## Abstract

The brain and gut are sensory organs responsible for sensing, transmitting, integrating, and responding to signals from the internal and external environment. In‐depth analysis of brain–gut axis interactions is important for human health and disease prevention. Current research on the brain–gut axis primarily relies on animal models. However, animal models make it difficult to study disease mechanisms due to inherent species differences, and the reproducibility of experiments is poor because of individual animal variations, which leads to a significant limitation of real‐time sensory responses. Organ‐on‐a‐chip platforms provide an innovative approach for disease treatment and personalized research by replicating brain and gut ecosystems in vitro. This enables a precise understanding of their biological functions and physiological responses. In this article, we examine the history and most current developments in brain, gut, and gut–brain chips. The importance of these systems for understanding pathophysiology and developing new drugs is emphasized throughout the review. This article also addresses future directions and present issues with the advancement and application of gut–brain‐on‐a‐chip technologies.

## Graphical abstract

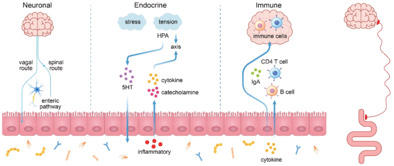

## 1 INTRODUCTION

The central nervous system organ, the brain, demonstrates remarkable complexity in the formation of memory, learning, and other cognitive capacities due to its inherent ability to self‐organize hierarchical designs at several spatial scales, from the molecular to the organismal level.^1^, ^2^ The World Health Organization states that conditions relating to the brain, such as different neurological and psychiatric disorders, have the greatest social burden of all diseases, accounting for 28% of the total, surpassing cardiovascular diseases and cancer.^3^ Mental illnesses are further exacerbated, especially under the influence of the COVID‐19 pandemic. The study of major brain illnesses is essential and difficult research in the field of brain science and technology for the future since the brain is thought to be the most significant organ in the human body and most likely the most complicated thing in the universe. There is now growing evidence that the development of neurological and psychiatric disorders is closely related to gut microbial dysbiosis.^4^, ^5^, ^6^, ^7^ In individuals with neuropsychiatric disorders, the microbial community composition, diversity, and distribution characteristics are often dramatically altered. It follows that many neuropsychiatric problems in humans are closely related to digestive problems, suggesting that the gut–brain axis also plays an important role in the cognitive functions of the brain.^8^, ^9^, ^10^, ^11^, ^12^

The brain–gut axis is a neuroendocrine‐immune signalling network system in which the brain is bidirectionally connected to the gut via the hypothalamic–pituitary–adrenal axis, the enteric nervous system (ENS), and the central nervous system (CNS).^6^ The brain and gut are sensory organs responsible for sensing, transmitting, integrating, and responding to signals from the internal and external environment.

As a result of this function, immune cells in the brain and intestines are constantly sensing environmental factors, which trigger responses that reflect the body's physiological status.^4^ For example, increased intestinal permeability may cause systemic immune dysregulation and lead to neuroinflammation.^13^ Moreover, while the intestinal epithelium blocks harmful foreign compounds from entering the circulation, the blood–brain barrier maintains chemical and physical balance, protecting the brain from harmful molecules and pathogens in the bloodstream.^14^ It has been demonstrated that certain secretions from intestinal cells (such as exosomes or enterotoxins) can travel to the blood–brain barrier via the bloodstream. They play a role in regulating inflammatory insults, inflammatory responses, and the maintenance of immune homeostasis through interactive signalling in the gut–brain axis.^15^, ^16^, ^17^ Thus, an in‐depth analysis of the brain–gut axis interactions is crucial for human health, and disease prevention, and control.

Currently, studies of the brain–gut axis primarily rely on animal models. However, due to inherent species differences, animal models are challenging to use in studying disease mechanisms. Additionally, experimental reproducibility is often poor due to individual variations among animals, which can significantly limit real‐time sensory response.^18^ Notably, the species‐specificity resulting from the genome and epigenome can also contribute to clinical trial failures. Therefore, there is an urgent requirement for a new in vitro model for studying the modulations of brain–gut axis interaction in clinical settings.

Organoids closely resemble the natural tissues or organs from which they are derived in terms of genetic profile, organization, and function. Therefore, they offer a promising research platform for developmental biology, disease modelling, drug screening, and cell therapy.^19^, ^20^, ^21^, ^22^, ^23^ Currently, organoid culture techniques have successfully cultured a variety of tissue‐like organs (e.g., brain, prostate, pancreas, kidney, etc.) with key physiological structures and functions.^24^, ^25^, ^26^, ^27^, ^28^, ^29^, ^30^, ^31^, ^32^, ^33^, ^34^ For example, Motallebnejad et al.^35^ created a 3D hydrogel model of the blood–brain barrier by embedding induced pluripotent stem cell‐derived brain microvascular endothelial cells and astrocytes in it. In recent years, some researchers have developed multi‐organ chips to simulate the interactions between the brain and other organs.^36^, ^37^, ^38^, ^39^ These platforms stimulate the brain and gut ecosystems in vitro, offering an innovative approach for conducting personalized therapeutic research on diseases. This enables a more accurate understanding of their underlying biological functions and physiological responses.

The development of gut‐on‐a‐chip, brain‐on‐a‐chip, and gut–brain‐on‐a‐chip systems and recent advances are reviewed. The role of these systems in the study of pathophysiology and in the development of new drugs will be highlighted. Current challenges and future perspectives in the development and exploitation of gut–brain‐on‐a‐chip platforms are also discussed.

## 2 COMMUNICATION MECHANISMS BETWEEN THE GUT AND THE BRAIN

### 2.1 Neural pathways of the brain–gut axis

**FIGURE 1:**
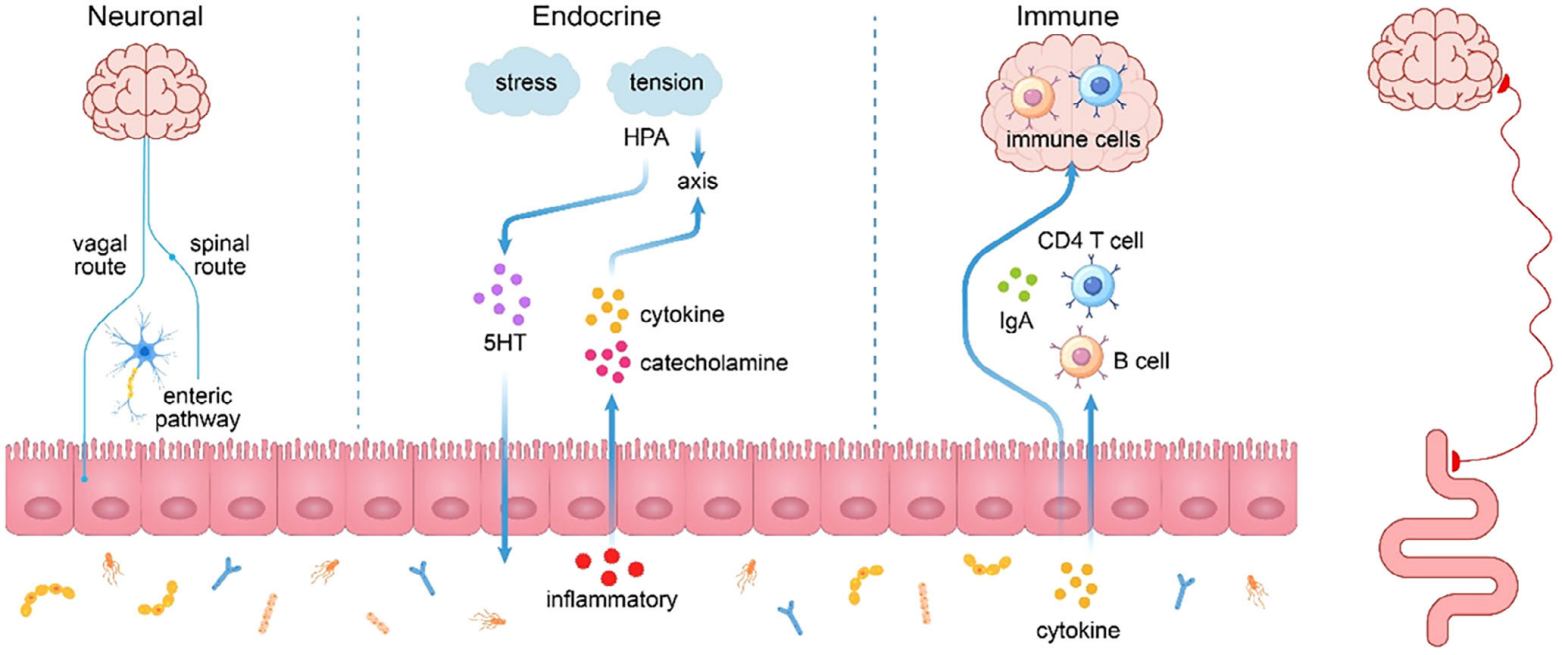
Schematic diagram of the three pathways of the brain–gut axis.

The complex interplay between the brain and gut relies on a well‐organized nervous system (Figure 1), divided into three main parts: the central nervous system, the autonomic nervous system (ANS), and the enteric nervous system.^40^ The autonomic nervous system transmits signals from the gut to the brain via the enteric, spinal, and vagal routes. Conversely, the central nervous system regulates the activity of gastrointestinal cells through the autonomic nervous system. The enteric nervous system, often referred to as the “second brain”, is composed of a vast network of nerve cells scattered throughout the intestinal wall, which autonomously govern the digestive tract's movements and secretions.^41^

Extensive research has been conducted on the vagus nerve, a component of the autonomic nervous system, due to its significant role in regulating inflammation as well as hunger, satiety, and stress responses.^42^, ^43^, ^44^ The vagus nerves originate from the brain, travel through the intestines, and reach the intestinal wall. Additionally, some vagus nerves are connected to the enteric nervous system through synapses, expanding the scope of information transmission.^45^ In animal studies, disruption of the vagus nerve associated with the gut has been found to affect neurological development in adulthood, stress, anxiety and fear‐related behaviours, and cognitive changes.^46^ All of this evidence suggests that the gut can communicate with the brain through neural interactions that influence cognition and behaviour.

### 2.2 Endocrine pathways of the brain–gut axis

The endocrine pathway of the brain–gut axis, known as the neuroendocrine pathway, regulates various activities through hormones or neuropeptides.^47^ The focus of this pathway is the hypothalamic–pituitary–adrenal axis (HPA axis), which regulates a wide range of daily activities, including digestive, sexual, and immune responses, as well as stress responses.^48^, ^49^

The hypothalamic–pituitary–adrenal (HPA) axis can influence intestinal motility, permeability, and the inflammatory response, thereby modulating intestinal function during digestion.^50^ CRH receptors in this pathway are distributed in both the brain and the gut. It is an important mediator of stress‐related alterations in gut function.^51^, ^52^ Stress and tension significantly modulate the HPA axis, stimulating the release of neurons and neuroendocrine signalling molecules (e.g., catecholamines, 5‐hydroxytryptophan, and cytokines), which can lead to gastrointestinal dysfunction. At the same time, the inflammatory response in the gut can also activate the HPA axis via cytokines and other factors, which in turn affect the brain. This is one mechanism that contributes to the low mood caused by inflammatory diseases such as irritable bowel syndrome.

### 2.3 Immune pathways of the brain–gut axis

The gastrointestinal tract has the highest number of immune cells in the body, and the immune system plays a crucial role in the brain–gut axis. The mucosal immune system provides the most direct specific immunity to the intestinal site. T cells and B cells in the mucosa, along with secretory immunoglobulin A (sIgA) secreted into the intestinal lumen after B cell activation, work together to provide localized mucosal immunity in the intestinal tract.^53^, ^54^ The central nervous system regulates the peripheral immune response through the HPA axis and the sympathetic nervous system. Meanwhile, the gut–brain axis involves various immune and non‐immune cells that form a complex network of interactions. This network modulates the inflammatory response of the brain and gut to external stimuli and transmits inflammatory signals.^4^, ^55^ The gut–brain axis regulates inflammation through the delivery of immune factors known as the systemic‐humoral pathway. Exogenous stimuli such as local infections, dysbiosis, or dietary antigens can lead to the release of gut‐derived inflammatory factors including IFN‐g, IL‐6, and TNF‐a, causing an increase in intestinal permeability and disrupting the blood–brain barrier, thereby activating local immune cells and triggering neuroinflammation.^14^ Additionally, in the pathway, inflammation‐induced hypothalamic–pituitary–adrenal (HPA) axis activity triggers the release of glucagon and corticosterone, which alter gut function.^55^ Furthermore, gut immune cells had the ability to directly impact neuroimmune homeostasis and brain responses to inflammation in the gut–brain axis, known as the cellular immune pathway. The activating inflammatory immune cells can then move to the CNS and exacerbate neuroinflammation in certain diseases like multiple sclerosis (MS) or Parkinson's disease (PD).^56^ Also, it has been determined that intestinal antigens can activate immunoglobulin A (IgA)‐secreting plasma cells and control gut microbial composition, while IgA^+^ plasma cells migrate in large numbers to the CNS to attenuate neuroinflammation in autoimmune diseases of the nervous system.^57^, ^58^ Finally, Inflammatory signals transmitted by neuronal pathways in the gut–brain axis can trigger a CNS response to restore homeostasis. It was reported that the inflammation‐mediated vagus nerves trigger CNS circuits and participate in HPA activation.^59^ Neurons like the dorsal root ganglion (DRG) neurons can signal inflammatory stimuli to the CNS to promote inflammatory pain,^60^ and modulate M‐cell function to prevent intestinal pathogenic infections.^61^ Whereas, stress‐induced output neurons can signal immune cells to suppress intestinal inflammation by inhibiting the release of inflammatory factors IL‐1β, IL‐6, IL‐18, and TNF‐α from macrophages.^62^

### 2.4 Microbiota–gut–brain axis

The gut microbiota plays a crucial role in the gut–brain network. It exerts significant influence on the brain–gut axis by regulating bidirectional communication between the gut and the central nervous system, forming the microbiota–gut–brain axis.^63^ Its regulatory functions are carried out through three highlighted major pathways: the neural pathway, the endocrine pathway, and the immune pathway.^64^

In the neural pathways, gut microbiota such as *Lactobacillus johnsonii*, *Lactobacillus rhamnosus*, and *Lactobacillus* (*L*.) *reuteri*, and their metabolites, have been shown to impact the neurons of ENS and engage with the afferent pathways of the vagus nerve to control sleep.^62^, ^65^, ^66^ Additionally, gut microbes influence neurodevelopment, cognition, and behaviour. For example, high‐fat or high‐carb diets are thought to be the main cause of obesity, and obesity also leads to the disruption of the diversity and stability of the gut microbiota.^67^ Diet‐induced gut flora, in turn, affects hypothalamic function, particularly inflammation and gliosis, by influencing vagal afferent signalling. This stimulation leads to the onset of obesity and subsequent obesity‐related complications. Futhermore, the gut microbiome has been reported to regulate the development, maturation, and function of microglia located in the brain.^68^

In the endocrine pathway, gut microbiota such as *Bifidobacterium adolescentis, Clostridiaceae*, *Turicibacteraceae*, *Morganella morganii*, and *Lactobacillus vaginalis* have the ability to directly or indirectly impact the synthesis of neurotransmitters and metabolites, including Melatonin (MT), GABA, Serotonin (5‐hydroxytryptamine, 5‐HT), and Histamine, which are involved in regulating the sleep‐wake cycle through the endocrine system.^69^, ^70^, ^71^, ^72^ Additionally, short‐chain fatty acids (SCFAs) produced by gut microbiota induce the secretion of gastrointestinal hormones like glucagon‐like peptide 1 (GLP1) and peptide YY (PYY) along the endocrine pathway, transmitting signals to the brain through systemic circulation or the vagus nerve pathway, thus impacting cognitive functions, memory, and emotional well‐being.^73^

In the immune pathway, immune factors originating from the gut can affect sleep through the humoral system and neural pathways. For example, intestinal microbiota‐mediated Interleukin 6 (IL‐6), lipopolysaccharides (LPS), and short‐chain fatty acids (SCFAs) can influence immune cell responses, thereby regulating sleep.^74^, ^75^, ^76^, ^77^ Additionally, the microbiota colonizing the gut directly influences the body's immunoregulatory effects of the body by promoting antigen secretion and regulating the production of various cytokines. In Alzheimer's disease, the integrity of the blood–brain barrier (BBB) is significantly compromised due to bacterial translocation and the release of pro‐inflammatory cytokines, triggering a series of neuroinflammatory reactions.^78^, ^79^, ^80^ Conversely, the immune system also exerts a regulatory and restrictive effect on the intestinal flora, maintaining the balance of microbial populations in the intestines by regulating and limiting their quantity and diversity.^81^ It can recognize and eliminate harmful microbial populations while supporting the growth and proliferation of beneficial microbial populations. Moreover, the immune system can establish a barrier in the intestines to prevent harmful microbiota from invading the intestinal mucosa.^82^, ^83^

## 3 ORGAN ON A CHIP (GUT–BRAIN ON A CHIP)

Studying the role of microbiota in neuropathy has been a challenge due to the absence of an in vitro model of the microbiota–gut–brain axis. This gap has prevented researchers from uncovering the potential mechanisms of change between microbiota and the nervous system. To improve the reliability of in vitro tools, bioengineering research is focusing on the use of advanced technical equipment and 3D engineering models. The “organ‐on‐a‐chip” microfluidic system, which combines lab‐on‐a‐chip technology with 3D organ culture, is a new frontier of in vitro models for bioengineering research.^84^, ^85^, ^86^, ^87^ Organ‐on‐a‐chip is a millimetre‐scale micro‐bioreactor that can be continuously perfused with culture media for long‐term in vitro culture. Organ‐on‐a‐chip technology not only recapitulates the smallest functional aspects of physiological and pathological states in tissues and organs but also helps to assess the efficacy of therapeutic agents. It reduces costs, increases yield, and reduces ethical concerns compared to animal models.

Organs‐on‐a‐chip have been utilized to model various organs and tissues in the body. These chips simulate the transportation of substances through microanatomical interfaces, such as the blood–brain barrier (BBB), and can even be used to culture intestinal microbiota. They are used to investigate the connection between microbiota and other organs. For instance, the co‐culture of Bifidobacterium bifidum and intestinal epithelial cells was conducted using dual‐channel intestinal microarrays to investigate the role of Bifidobacterium bifidum in inflammatory bowel disease.^88^ First, symptoms of the intestinal disease were induced on the microarrays by treatment with tumour necrosis factor‐alpha and lipopolysaccharide. Second, steep gradients can be created in the intestinal epithelium by varying the size of the microchannels. This can be done by blocking oxygen permeation through the polydimethylsiloxane (PDMS) layer at specific sites and by controlling the flow of hypoxic/aerobic media. This model confirmed that by preventing disruption and promoting the repair of damaged intestinal epithelial cell monolayers, bifidobacteria help to stabilize the intestinal epithelial barrier.

The main technical challenge is to connect individual organ‐on‐chip devices to simulate the passage of microbiota‐secreted neurotoxins across different body systems and microanatomical barriers to investigate the microbiota–brain axis hypothesis. Moving from a microbiota–brain microarray approach to a more complex “multi‐organ microarray” strategy appears to be a promising alternative to animal models. In the following paragraphs, we shall discuss the most recent cell culture systems for microbiota, gut, and brain modelling.

## 4 MICROBIOTA–GUT–BRAIN MODELLING

### 4.1 Gut‐on‐a‐chip

Gut microarray systems provide a new and powerful in vitro platform for the study of the physiology, pathology, and pharmacology of the human gut. The understanding and treatment of intestinal diseases, such as inflammatory bowel disease (IBD) and colorectal cancer, will benefit from these gut microarray systems. The development of gut microarrays will also help to advance personalized medicine and drug screening technologies. In the following section, the role of these systems in the study of pathophysiology and drug development is highlighted, with an overview of the development and recent advances in gut‐on‐a‐chip technology (Table 1). The role of these systems in the study of pathophysiology and drug discovery will be highlighted with an overview of the development and recent advances in gut‐on‐a‐chip technology.

**TABLE 1:** Gut‐microbe‐on‐a‐chip.

Target disease	Chip material	Micro‐organisms	Cell type	Application	References
Inflammatory bowel disease (IBD)	OrganoPlate		Caco‐2 cells	High throughput disease modelling and drug discovery	^114^
Shigella infection in the gut	PDMS	*S. flexneri*	Caco‐2/TC7 cells	Biomedical research of host pathogens interactions in a more physiological environment.	^115^
/	PDMS	Bacteroides fragilis; a fresh gut microbiome	Caco2 intestinal epithelial cells and human intestinal microvascular endothelial cells (HIMECs)	Study host−microbiome interactions	^116^
/	PDMS	Cryptosporidium parvum	Mouse intestinal crypts	Construction of open‐accessible epithelial cells with in vivo anatomical resemblance from primary stem and progenitor cells	^117^
SARS‐CoV‐2 induced intestinal responses	PDMS	SARS‐CoV‐2	Caco‐2, HUVECs, Human peripheral blood mononuclear cells	Accelerate COVID‐19 research	^118^
/	PDMS	*Lactobacillus rhamnosus, Bifidobacterium longum*	Caco‐2 cells, hISEMFs (human intestinal subepithelial myofibroblasts)	Develop an immune‐responsive Microbiota‐human Intestine axis model	^88^
Necrotizing enterocolitis	PDMS	Intestinal bacteria isolated from a patient with severe	Human neonatal enteroids from infants undergoing surgical resection	Facilitates comprehensive analysis of the pathophysiology of NEC using precious clinical samples	^119^
Inflammatory bowel disease	PDMS	*Bifidobacterium bifidum*	Caco‐2 cells	Mimic key physiological and pathological aspects of IBD, and screen large‐scale of probiotics for specific treatment of IBD.	^84^
Colorectal cancer	PDMS	*Bacillus bacteria or lipopolysaccharide* (*LPS*)	A human colorectal carcinoma cell line (HCT116)	Simulating the Effect of Gut Microbiome on Cancer Cell Growth	^86^
Ulcerative colitis, Crohn's disease, colorectal cancer or normal donors	PDMS	*Green fluorescence protein* (*GFP*)*‐expressing Escherichia coli*	Caco‐2 cells or intestinal organoid epithelial cells	Re‐creating the 3D epithelial structure to building in vitro intestine models	^87^
Inflammatory bowel disease	PDMS	*Bifidobacterium bifidum*	Caco‐2 cells	A host–microorganism co‐cultured Gut Chip model with controlled oxygen gradients	^84^
	Special plate (TL6)		Caco‐2 and HT29 cells; Human hepatocytes (lot: RAS)	The quantitative in vitro pharmacokinetic study of mycophenolate mofetil	^85^
Inflammatory bowel disease	PDMS, Gelatine porous microscaffolds	*Intestinal commensal microbiota* (*Lactobacillus rhamnosus and Bifidobacterium longum*)	Caco‐2 cells, Human intestinal subepithelial myofibroblasts (hISEMFs), Peripheral blood mononuclear cells (PBMCs)	The recapitulation of the mucosal‐associated microbiota‐cell interactions into the complex microenvironment in both physiological and pathological conditions.	^88^

To date, gut microarrays have been developed primarily for the study of basic gut functions and how they are affected by environmental conditions, drugs, and other factors. For example, Shin et al.^87^ have developed an in vitro intestinal model using a combination of microfluidic microarrays and transwell for robust induction of intestinal morphogenesis. This study provides detailed methods for the fabrication of a gut‐on‐a‐chip microfluidic device and a hybrid chip with a transwell insert for the in vitro culture of intestinal epithelial cells on either a porous PDMS membrane or the polyester membrane of a transwell insert and the induction of 3D morphogenesis. Physiologically relevant shear stresses and mechanical movements, which do not require complex cell engineering or manipulation, are used in this in vitro morphogenetic approach. In another study, a controlled oxygen gradient on a two‐channel chip was developed in an intestinal organ chip. This study aimed to investigate the therapeutic effects of *bifidobacterium bifidum* on inflammatory bowel disease (IBD).^84^ To create an oxygen concentration gradient across the entire intestinal epithelium, the researchers modified the size of the microchannel, blocked oxygen permeation through the PDMS layer at certain points and regulated the flow of hypoxic/aerobic media. Tumour necrosis factor‐alpha and lipopolysaccharide were used to induce IBD symptoms on the chip. The oxygen requirements of aerobic human intestinal epithelial cells (Caco‐2) and anaerobic intestinal bifidobacteria (B. bifidum bifidum) were successfully met by the stable oxygen concentration gradient in the study. By colonizing the intestinal microarray with Bifidobacterium, it was shown that Bifidobacterium can improve the stability of the intestinal epithelial barrier (IEB). This simple yet robust design approach of the microfluidic device represents the first step toward the creation of a multifactorial IBD platform. It could be used for disease modelling, high‐throughput drug screening, and personalized medicine.

In addition, the gut‐on‐a‐chip model can be a tool for the study of gut–immune interactions. Recently, an immunoreactive human microbiota–gut axis chip platform was developed.^88^ By simulating the intestinal microenvironment, the platform replicated the structure and vertical morphology of the intestinal microflora. It mimics the anaerobic conditions of the human intestinal microenvironment by establishing a physiological oxygen gradient across the thickness of the human small intestine from the serosal membrane to the side of the intestinal cavity. This environment includes a reactive extracellular matrix (ECM) and a variety of immunomodulating mediators released by different cell populations. Highlighting the role of this probiotic in the anti‐inflammatory process, lipopolysaccharide (LPS)‐induced inflammation was found to alter the microbiome and induce Bifidobacterium longum proliferation using this intestinal organoid chip. This study demonstrated for the first time the indirect role of microbiota in immune‐responsive MihI‐oC matrix remodelling, focusing on collagen fibre orientation and ECM remodelling. Meanwhile, the study demonstrated the role of microbiota in enhancing gastrointestinal immunity and fighting infectious diseases by analysing the release of key immune mediators (reactive oxygen species (ROS), pro‐ and anti‐inflammatory cytokines) after inflammatory stimulation.

In addition, integrating other organs to build multi‐organ models is an important prerequisite for developing in vitro systems that can accurately replicate biological responses. For example, to study intestinal permeability, metabolism (intestinal and liver), and the potential interplay between these processes, a gut‐liver organ‐no‐a‐chip system was used using CaCo_2_ cells in co‐culture with HT29 cells in the intestinal compartment and single donor primary hepatocytes in the liver compartment.^85^ Since both the gut and the liver contribute to the metabolism of the prodrug mycophenolate mofetil, it was tested for quantitative evaluation in the gut‐liver organ‐on‐a‐chip (OoC) model. In the apical, basolateral, and liver compartments of the gut, the conversion of mycophenolate mofetil to the active drug mycophenolic acid and its subsequent metabolism to a glucuronide metabolite were evaluated over time. Clearance and permeability parameters for the prodrug, active drug, and glucuronide metabolite were estimated using mechanistic modelling of experimental data. The integration of gut‐liver OoC data within silico modelling allowed the investigation of the intricate interplay between intestinal and hepatic processes, a feat not possible with conventional single‐tissue in vitro systems. Robust experimental design and estimation of in vitro pharmacokinetic parameters were facilitated by a comprehensive evaluation of the mechanistic model, including structural model and parameter identifiability, and global sensitivity analysis. This study suggests that similar methodologies could be applied to other multi‐organ microphysiological systems used to study drug metabolism, or wherever quantitative knowledge of the changing drug concentration over time allows a better understanding of biological effects.

### 4.2 Brain‐on‐a‐chip

The brain, a complex organ of the human body, can be artificially constructed in vitro to construct bionic brain organoids, offering a promising platform for personalized medicine (Table 2).^89^ Especially in recent decades, due to the significant increase in the incidence of neurodegenerative diseases (NDD), the development of new biological platforms to study disease progression and drug efficacy has been of great interest. The blood–brain barrier (BBB), an organ‐on‐a‐chip (OoC) platform, mimicking the performance of the brain barrier, will enable a better understanding of NDDs.

**TABLE 2:** Brain‐on‐a‐chip.

Chip material	Cell	Matrix	Culture time	Application	Ref
PDMS	Human PSC‐derived cerebral organoids	Decellularized human brain extracellular matrix (BEM)	75 days	Invitro model and drug development of human brain diseases	^92^
PDMS	Human neural stem cells (NSCs), brain endothelial cells (ECs) and pericytes (PCs)	BEM‐mimetic hydrogel	5 days	Cryptococcus neoformans brain infection model	^120^
PDMS	Human primary astrocytes, Transformed human microglial cell line (HMC3, ATCC, CRL‐3304), Human primary BMECs, Human brain vascular pericytes, hEPCs, hBMSCs, hAMSCs	Basement membrane extract (BME) hydrogel	18 days	Evaluation of the restorative potential of stem cell therapies for ischaemic stroke	^91^
PDMS	Commercial human iPSC‐derived dopaminergic neurons, Primary human astrocytes, Brain microvascular endothelial cell	–	8 days	Enable research into the dynamics of cell–cell interactions in human synucleinopathies and serve as a testing platform for target identification and validation of novel therapeutics	^90^
PDMS	hBMECs, human neural progenitor cells	Collagen type I	–	Investigate the function and molecular changes of the brain endothelium and neuronal cells upon hyperglycemic or in glucose‐recovery condition even in the inflammatory condition.	^121^
PDMS	HCMEC, human cerebral microvascular endothelial cells	Extracellular matrix (ECM)	10 days	Investigate neuroinflammation	^122^
PDMS	ReN VM human neural (ReN) cells	Collagen type I, Matrigel, laminin	8 days	Assess Aggravated Neurodegeneration via Brain Endothelial Cells upon Exposure to Diesel Exhaust Particles	^123^
Poly (methyl methacrylate) (PMMA), PDMS	Organotypic entorhinal hippocampal tissue cultures prepared from mouse pups at 3 to 5 days post‐birth (P3 P5) from different mouse lines	–	18 days	Investigate brain stimulation mechanistic	^124^
PDMS	TNBC MDA‐MB‐231, the BR‐derived line MDA‐MB‐231‐BR‐GFP, Her2þ breast cancer JIMT‐1, and the BR‐derived line JIMT‐1‐BR cells	–	9 days	Investigate brain metastases	^125^
PDMS	hiPSCs	–	60 days	Recapitulate inter‐regional and intercellular interactions in human brain in vitro	^126^

**FIGURE 2:**
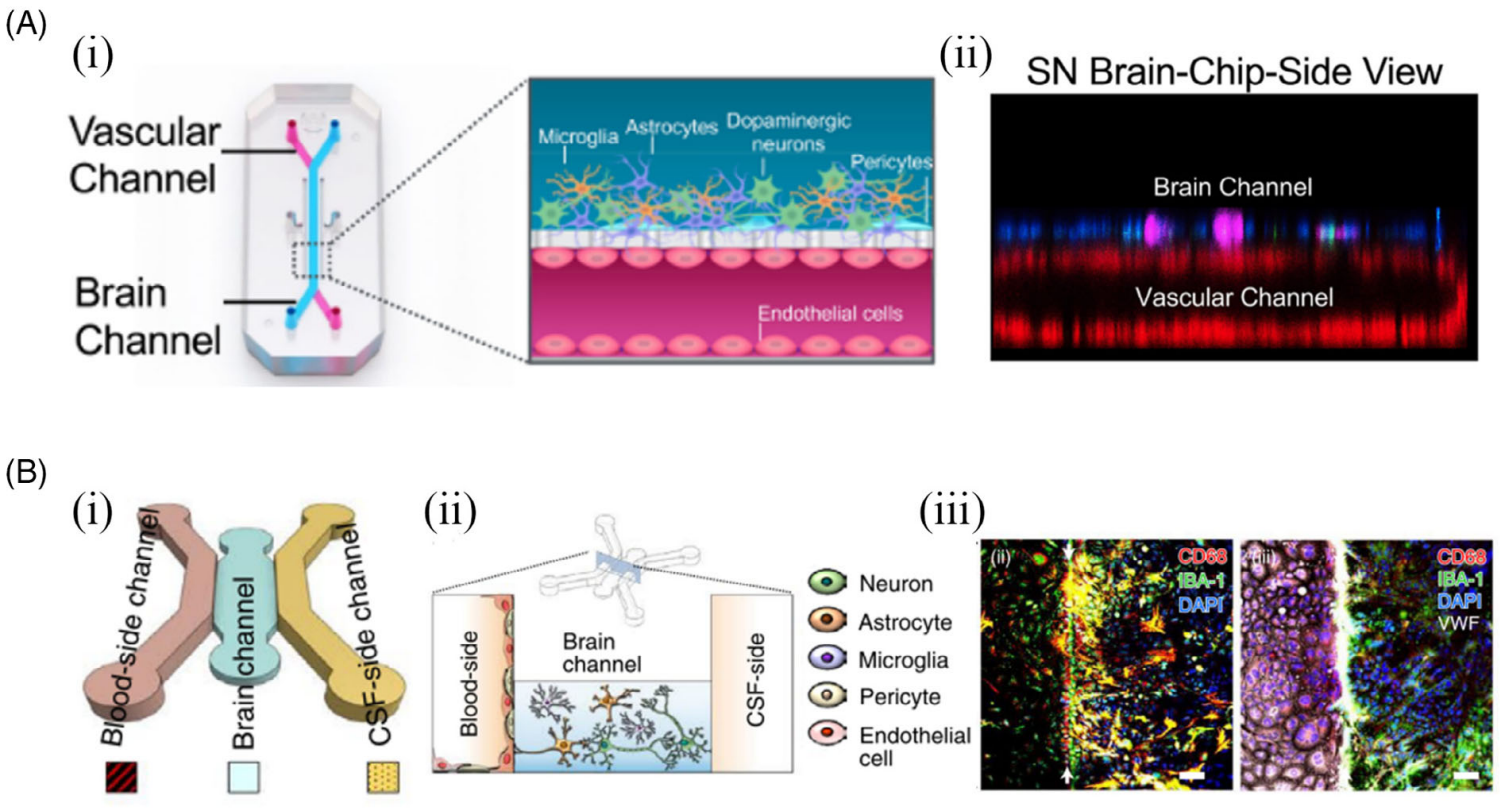
Brain‐on‐a‐chip and its applications. (A) A human Brain‐Chip representative of the substantia nigra area of the brain. Adapted with permission from Reference [90]. (i) Schematic representation of the Substantia Nigra Brain Chip showing a two‐channel micro‐engineered chip with iPSC‐derived brain endothelial cells cultured on all surfaces of the lower vascular channel. In addition, the top surface of the central horizontal membrane in the upper brain channel contains iPSC‐derived dopaminergic neurons, primary human brain astrocytes, microglia, and pericytes. (ii) 3D reconstruction of the confocal z‐stack, showing the organization of all five cell types in the SN chip. (B) A microfluidic chip representative of the blood–brain barrier. Adapted with permission from Reference [91]. (i) Chip design schematic. (ii) Brain microvascular endothelial cells (BMECs) are among the vascular cells. Spatial distribution of NVU constituent cells in the chip. (iii) Microglia (stained green by IBA‐1) showed upregulated expression of CD68 (red), a pro‐inflammatory microglial marker, and appeared yellow in the sample without endothelium (white arrows indicate the hydrogel border).

Recently, a human brain chip representing the substantia nigra region of the brain has been developed using organ‐on‐a‐chip technology to study the mechanism of blood–brain barrier disruption in Parkinson's disease caused by the accumulation of alpha‐synuclein (αSyn).^90^ This organ‐on‐chip (Figure 2) replicates the vascular‐neuronal interface and will be colonized with human iPSC‐derived brain endothelial cells, pericytes, astrocytes, microglia, and dopamine neurons. The ability of the nigrostriatal brain chip to respond to abnormal alpha‐synuclein (αSyn) aggregation will be confirmed by mimicking the exposure state of abnormal alpha‐synuclein (αSyn) aggregation (Figure 2A). This model will serve as a platform for the identification and validation of drug targets and the evaluation of therapeutic efficacy in Parkinson's disease and other synucleinopathies. Another study reported developing and applying a functional neurovascular unit on a microfluidic chip as a microphysical model of ischaemic stroke (Figure 2B).^91^ This model helped to understand how the blood–brain barrier works and how therapeutic stem cells interact with host cells, including human brain microvascular endothelial cells, pericytes, astrocytes, microglia, and neurons. The infiltration of different candidate stem cells and the expression levels of genes associated with post‐stroke pathology were monitored using this model. It was found that each type of stem cell was involved in neural repair by facilitating endogenous repair rather than direct cell replacement. Furthermore, the restoration of synaptic activity did not involve neuronal regeneration, but rather the restoration of structural and functional integrity of the neurovascular unit. To systematically analyse the neurorestorative behaviour of different stem cell types currently being tested in clinical stroke treatment, this model serves as a reliable screening test platform. It can be readily applied to a wide range of other vascular disease studies.

As brain models, including the newer brain organoids, become larger, brain‐on‐a‐chip designs also appear to be evolving into macroscale devices. Neutrospheres grown in flow produced more robust and elaborate neuronal networks than neutrospheres grown in static conditions. This suggests that the interstitial level of slow, diffusion‐dominant flow influences the continuous supply of nutrients and oxygen, the transit of cytokines, and the removal of metabolic waste. For example, a microphysiological stroke system has been developed to test the neural repair capacity of stem cell therapy.^91^ This stroke model used human cells and provided a 3‐D environment like the one found in vivo, which mimics the natural interplay between therapeutic stem cells and the neurovascular units (NVUs). This model could serve as a reliable screening test bed for the systematic analysis of the neural repair behaviour of different types of stem cells that are currently being tested in the clinical treatment of stroke. Another study by Cho et al^92^ demonstrated that the functional and structural maturation of human brain organoids can be promoted and enhanced using a brain extracellular matrix microfluidic device for brain organoid culture.

**FIGURE 3:**
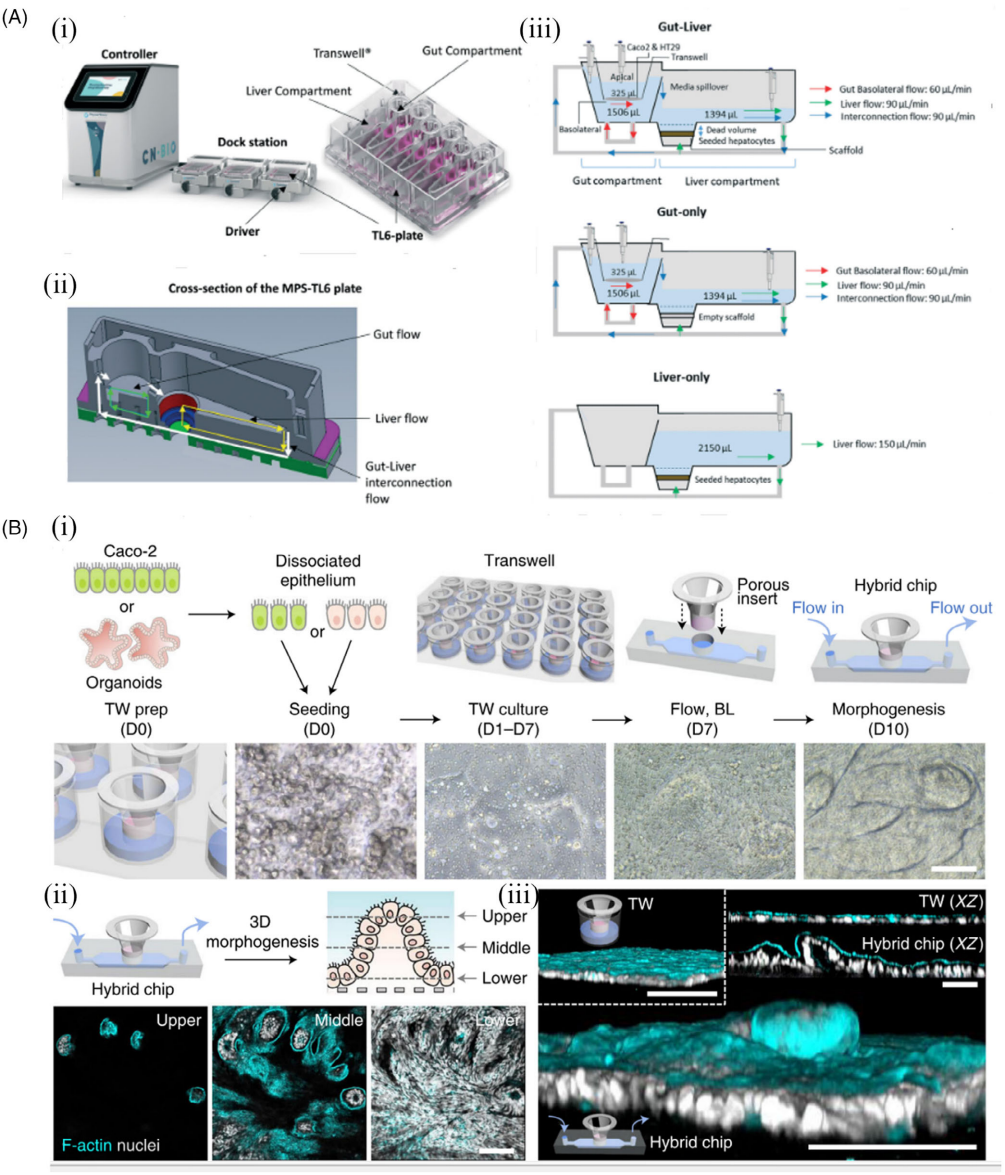
Gut on a chip and its applications. (A) Presentation of the Physio Mimix micro‐physiological systems (MPS) for the gut and liver. Adapted with permission from Reference [85]. (i) The system consists of a control machine with a set of pumps and a touch screen for user interaction. (ii)The main components of the system include a gut compartment with a transwell separating the apical and basolateral sides of the gut and a liver compartment seeded with hepatocytes. Three independent flow systems: Gut basolateral circulation, liver circulation, and interconnection flow. (iii) Graphs of borehole, media flow, and sampling points for all three test facilities. (B) Characterization of the 3D morphogenesis of epithelial cells in a transwell insertable hybrid chip. Adapted with permission from Reference [87]. (i) Hybrid chip workflow for 3D intestinal morphogenesis using Caco‐2 cells and organoids. Epithelial cells are seeded in a Transwell insert, cultured, then incorporated into a hybrid chip to create 3D layer. Micrographs show organoid epithelial cells from normal donor. (ii) The 3D morphogenesis of organoid epithelial cells can be facilitated by the hybrid chip. Confocal views at different Z‐positions show distinct morphological features. F‐actin (cyan), nuclei (grey). (iii) Fluorescence confocal images show 3D oblique views of the organoid‐derived epithelial cells cultured in a static transwell well (TW; inset within a white dashed box) compared to a hybrid chip (largest full image), showing the 2D and 3D morphology, respectively.

In addition, researchers have been developing 3D‐printed in vitro brain models to address the lengthy prototyping time and the absence of standardized experimental procedures in organ‐on‐a‐chip fabrication (Figure 3).^93^, ^94^, ^95^ 3D printing enables the creation of in vitro models of the central nervous system (CNS) using various materials, including living cells, to develop biologically active 3D structures along the z‐axis of the CNS (Figure 3A).^96^, ^97^ 3D‐printed in vitro models can be custom‐designed to accurately replicate in vivo neural tissue, ensuring consistency during clinical research and drug screening. Utilizing the advantages of 3D printing and microfluidic organ chips, it is expected that bionic brain chips can be constructed in vitro to replicate physiological and pathological processes observed in vivo, facilitating translational research.

To better understand the underlying pathology of traumatic brain injury, degenerative diseases, brain tumours, inflammation, infection, and other brain disorders, the development of in vitro brain models is an important strategy (Figure 3B).^98^, ^99^, ^100^ Although researchers are constantly updating and improving organoid technology, the gap between human brain organoids and the real human brain still exists and is recognized as an important scientific challenge in the field. Currently, human brain‐like organs are far less complex than real brain tissues in terms of structure and function. This is evident in the difference in size between the two, the absence of functional blood vessels, and the lack of neuroimmune cells in human brain‐like organs cultured in vitro.^89^ All these problems still need to be explored by researchers. We believe that shortly, with the efforts of scientists worldwide, a solution to the problem will enable the creation of a human brain organ that closely resembles the real human brain.

### 4.3 Gut–brain‐on‐a‐chip

**FIGURE 4:**
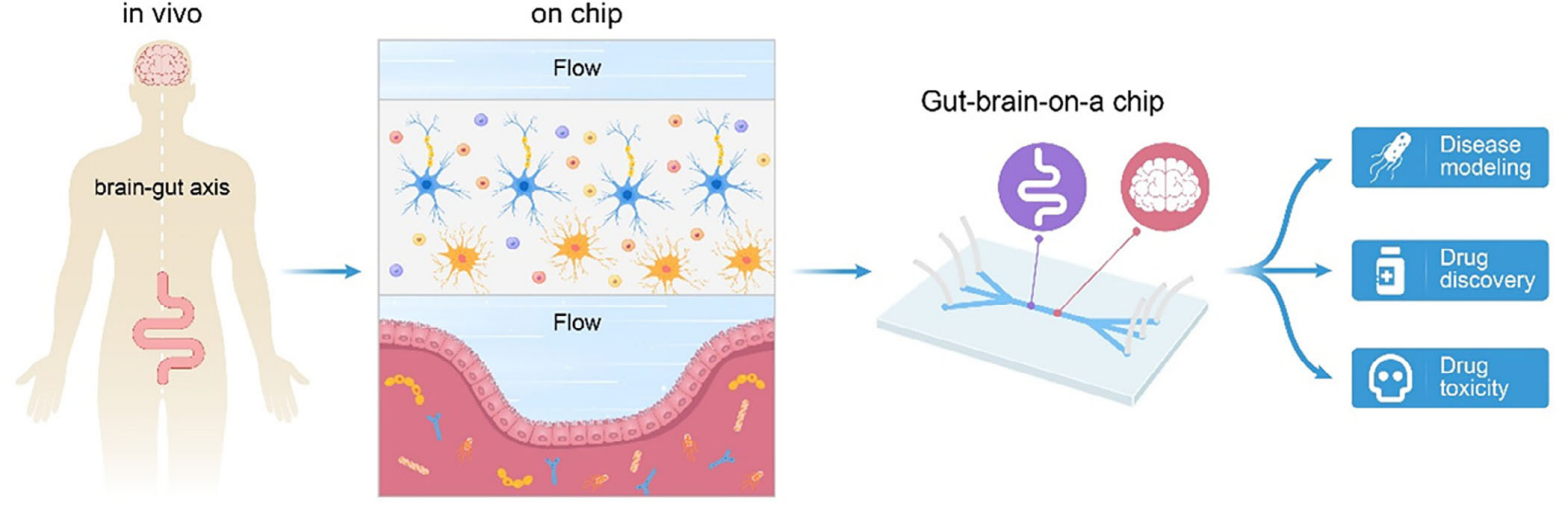
Schematic diagram of brain–gut‐on‐a‐chip.

It has been known for decades that the brain and gut need to communicate with each other to function. Gut–brain signals control basic functions and are linked to the development of complex neurological disorders, scientific studies have shown.^101^, ^102^ In addition, scientists have been investigating how this axis affects human health using in vitro model systems (Figure 4 and Table 3).

**TABLE 3:** Gut–brain‐on‐a‐chip.

Organ	Classification	Material	Cell	Application	Ref
Gut–brain	Microfluidic	Poly (ethylene glycol) (PEG) gel	Adult rat hippocampal progenitor cell (AHPC) neurosteroids	Investigate the interactions between microbiota and brain	^101^
Microbiota–gut–brain	Microfluidic		Microbiota, macrophages, lymphocytes, neurons, astrocytes, and microglia	Evaluate the impact of intestinal microflora on neurodegeneration in AD and/or PD and other neurodegenerative disorders	^105^
Gut–brain	Microfluidic	Polyester membranes, PMDS	Caco‐2 human gut epithelial cell line, bEnd.3 murine brain endothelial cell line, and hBMECs primary human brain microvascular endothelial cells	Assessed the transport of exosomes	^104^
Gut–brain	Microfluidic	PDMS	Caco‐2 cells, *Lactobacillus casei*, *Lactobacillus plantarum*, the human iPSC WTC cells	Evaluate the impact of microbial‐derived metabolites and exosomes on neurodevelopment and neurodegenerative diseases	^108^
Microbiota–gut–brain	Microfluidic	COLL‐PEG2000 gels, COLL‐PEG3350 gels	Neuroglioma H4 cells	Advanced engineered models representing brain features in Alzheimer's disease	^102^
Gut–brain	Microporous cell culture membrane	Carbon nanotube (CNT), a porous polyethylene track‐etched (PETE) membrane PDMS	RIN14B rat islet cell line	Detection of 5‐HT at physiological concentrations	^109^
Gut–brain liver	Pumping platform with integrated fluid channels		Colon organoids HC176, human monocyte‐derived dendritic cells, macrophages, human primary hepatocytes, Kupffer cells, neurons, astrocytes, and microglia, T and T helper 17 cells	Study gut‐liver‐cerebral interactions in the context of Parkinson's disease (PD)	^110^

**FIGURE 5:**
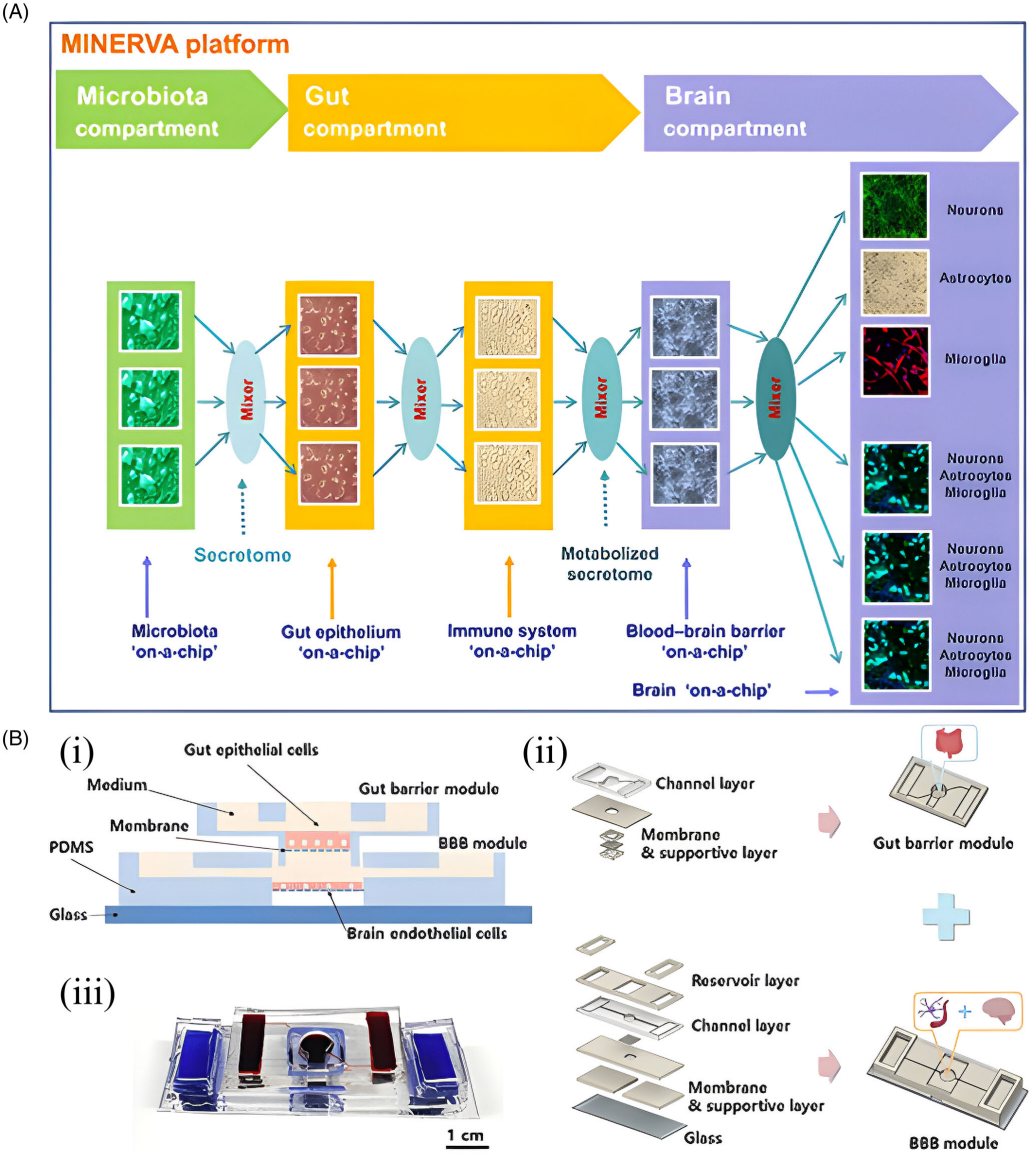
Structural design and application of gut–brain chip platform based on microfluidic technology. (A) MINERVA is a microfluidic Microbiota–Gut–Brain‐on‐a‐chip platform to study the Microbiota–Gut–Brain Axis in neurodegeneration. Adapted with permission from Reference [105]. MINERVA combines five miniature sensing, optically accessible organ‐on‐a‐chip devices, continuously connected by microfluidic tubing, and divided into three compartment modules. Gut microbiota compartment (green): Features a miniaturized microfluidic device hosting microbial cultures and soluble molecules of the microbiota, collectively referred to as the “secretome.” Intestinal compartment (yellow): Features two sets of organoids, a two‐dimensional culture system for intestinal epithelium and a culture system for immune system cells such as macrophages and lymphocytes, which are coupled to mimic parts of the immune system. Brain compartment (purple): The presence of two sets of organoids, a 2D culture system that mimics the BBB, and 3D conditions to cultivate major brain cells associated with neurodegenerative diseases, such as neurons, astrocytes and microglia. (B) Design of a microfluidic Gut–Brain‐on‐a‐chip. Adapted with permission from Reference [104]. (i) The assembled chip consisting of two barriers: Gut barrier (upper part) and BBB (bottom part). (ii) Schematics of two module which are gut barrier module (upper part) and BBB module (bottom part). (iii) Image of the assembled chip.

The gut–brain axis (GBA) microfluidic system is a valuable model for in vitro simulation of the gut–brain axis, with several advantages such as modularity and high throughput^103^, ^104^ (Figure 5). It simulates the bidirectional communication network between the gut and the central nervous system. This system is crucial for researching diseases and pathological mechanisms associated with the gut–brain axis.^103^ In 2017, the European Research Council (ERC) funded the first project on a bionic platform named MINERVA.^105^, ^106^ This platform, designed to explore the microbiota's role in neurodegenerative diseases like Alzheimer's, features a microbiata–gut–brain multi‐organ microfluidic microchip (Figure 5A). It was divided into three modules: microbiota, gut, and brain compartments. The microbiota module cultivates gut microbiota that release soluble molecules. These are then transported through microfluidic tubing to the gut compartment, which accommodates epithelial cells and immune system cells, including macrophages and lymphocytes. The brain compartment fosters the primary brain cells linked to neurodegeneration, such as neurons, astrocytes, and microglia. And its multi‐module layout allows for scalability in future research endeavours.^105^ Additionally, Min‐Hyeok Kim et al.^104^ developed a modular microfluidic chip for the GBA by co‐culturing intestinal epithelial cells and brain endothelial cells (Figure 5B). This chip includes microfluidic channels to simulate the formation of the intestinal epithelial barrier and the blood–brain barrier (BBB). The authors observed similar responses on this chip to the known microbial byproduct‐mediated interactions between the gut epithelium and the BBB, and explored inter‐organ communication between the gut and the brain with labelled exosomes.^104^ Hence, the GBA microfluidic chip can serves as a valuable platform for exploring the distribution and tropism of extracellular vesicles as drug carriers in vivo. It offers substantial technical support for in vitro investigations into the potential role of extracellular vesicles in the GBA and associated mechanisms.^107^ A study highlighted the impacts of gut microbe‐derived metabolites and extracellular vesicles on neurodevelopment and neurodegenerative conditions by developing a gut–brain axis microfluidic chip system containing neurons from human induced pluripotent stem cells (iPSCs). In vitro experimentation revealed that microbiota‐derived metabolites and exosomes travelling through the gut–brain axis could potentially trigger neural differentiation and enhance the expression of synapse‐associated proteins in neurons.^108^

**FIGURE 6:**
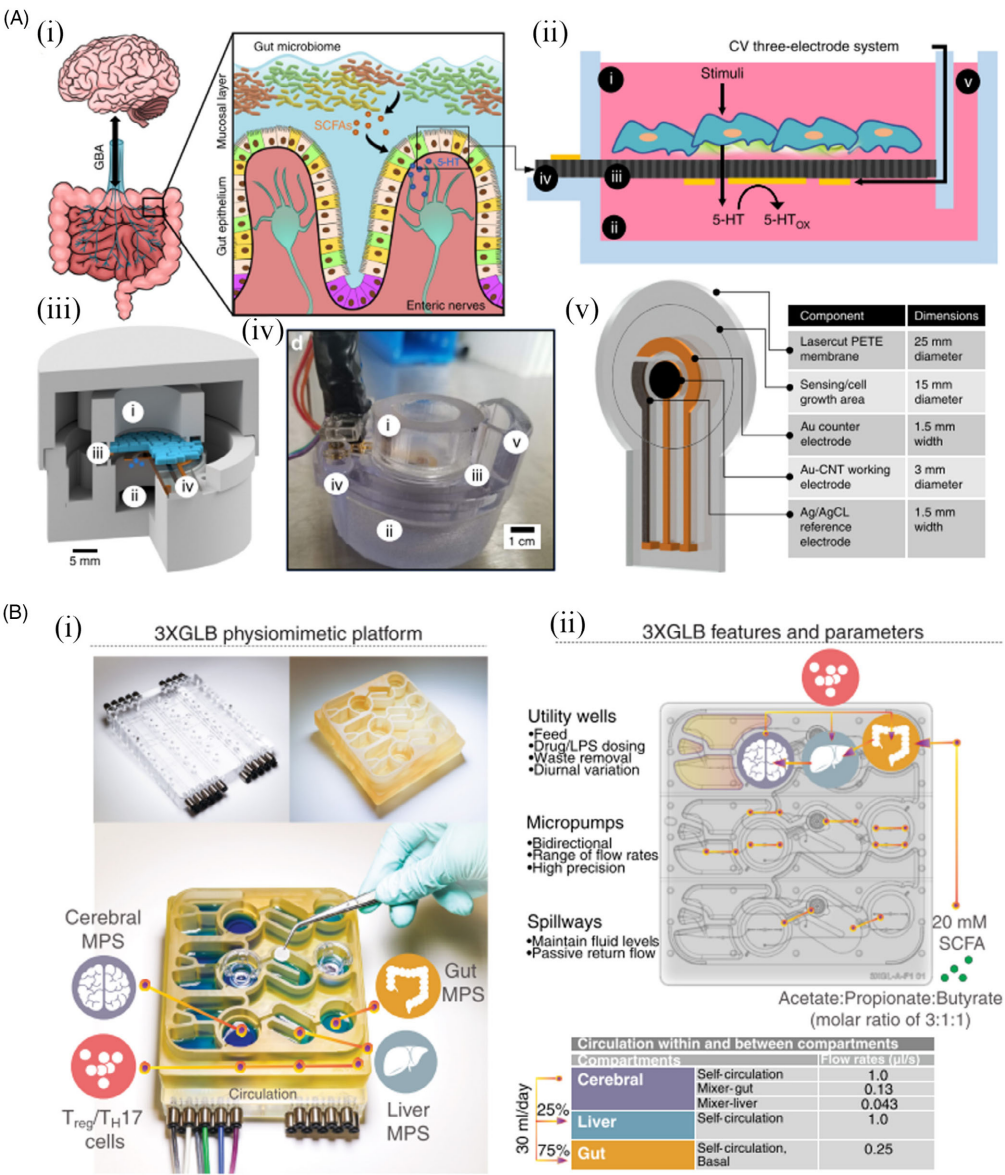
Experimental studies based on brain–gut‐on‐a‐chip. (A) Characterization of a gut–brain‐on‐a‐chip platform with integrated electrodes. Adapted with permission from References [109]. (i) Diagram of the physiology of GBA (left). Diagram of the luminal stimulation of the enterochromaffin cells in the intestinal epithelium and the subsequent basolateral secretion of 5‐HT for the activation of the enteric nerves (right). (ii) Schematic illustrating how the platform detects 5‐HT from cultured intestinal cells. 3D‐printed transwell system with the top chamber, bottom chamber, porous PETE membrane, integrated electrodes, external contacts, and fluid inlet channel. Cyclic voltammetry (CV) electrodes are positioned under the porous membrane to separate the cells from electronics. (iii) Conceptual CAD diagram showing the 3D arrangement of the chambers that sandwich the membrane integrated with the electrodes. (iv) The image shows a 3D‐printed platform with pogo‐pin connections at the contact pads. These pads were used for testing in this article. (v) CAD image of the porous membrane manufactured using the CV three‐electrode system. (B) A human gut‐liver‐brain‐chip representative for studies of neurodegenerative diseases. Adapted with permission from Reference [110]. (i) Acrylic airfoils (top left), monolithic polysulphone airfoils (top right) and 3× Gut–Liver–Brain (3×GLB) platform (bottom). (ii) The 3×GLB platform consists of pneumatic and fluid plates with an interposed polyurethane elastomer membrane forming a pump manifold with integrated fluid passages. The platform allows for a three‐way interaction in three replicates, where the central liver‐specific MPS can be fluidically connected to two additional MPS based on Transwell technology. (iii) Plan view, showing fluid and pump characteristics and operating conditions.

In addition to the microfluidic, researchers designed an experimental gut–brain system in a petri dish integrating with microporous cell culture membrane to identify molecules and signalling pathways that the gut and brain rely on to communicate^109^ (Figure 6). This system has a separate compartment for the ‘mini gut’ made up of endothelial cells and a separate compartment for the “mini‐brain” made up of crayfish nerves. The fluid connection between the two chambers facilitates the movement and monitoring of signalling molecules (Figure 6A). The system has identified the neurotransmitter serotonin as one of the keys signalling molecules that play a key role in gut–brain signalling. Furthermore, the system's sensors indicate that the neurotransmitter (5‐hydroxytryptamine) is effectively transferred from the endothelial surface to the underlying endothelial layer. Using the crayfish model, this system mimics the natural electrophysiological responses observed in animal studies. Without the need for invasive manipulation of humans or animals, it allows, for the first time, simultaneous real‐time in vitro monitoring of signals between two tissues of the gut–brain axis. Recently, researchers have developed a human gut‐liver‐brain physiological simulation system using a microphysiological system (MPS) (Figure 6B).^110^ This in vitro platform connects brain cells to tissue models of the colon and liver, enabling the flow of immune cells and nutrients (including short‐chain fatty acids (SCFA)) through the channels between them. This well‐established conceptual and experimental model of immunometabolic cross‐talk for sustained and long‐term interorgan system interactions is an important advance in the simulation of the human gut–brain axis in the context of in vitro neurodegeneration.

Hence, the GBA chips hold significant potential for learning the influence of the gut–brain axis on associated diseases. Additionally, it offers a robust platform for testing therapies. However, there are many technical challenges in the developing multi‐organ microarrays, leading to the fact that few studies have been reported. The GBA chips, as a multi‐organ chips, must meet specific physical constraints, considering organ size, flow rates for each module, and overall medium volume to achieve physiological relevance. It imposes higher demands on chip design and fabrication.^111^ Meanwhile, it is a challenging technical problem to precisely replicate the physical and chemical microenvironment of in vivo tissues and organ dynamics in the GBA chips.^112^ Moreover, the GBA chips, being more complex than single‐organ chips, often present greater difficulties and higher costs in fabrication.^113^

## 5 CONCLUSION

The gut–brain axis has become a significant research focus in the field of intestinal science. The development of relevant models is crucial for studying the communication mechanisms between the gut and the brain. The development of dynamic cell culture systems and in vitro models that mimic human organs has been enabled by advances in microfluidics and micromachining. Nowadays, preliminary results of in vitro construction of brain–gut axis models are available. These microarray systems are useful tools for the prediction of drug toxicity and for the in vitro modelling of different disease states in the brain. They are expected to improve healthcare by making drugs more accessible and less problematic, reducing the incidence of unpredictable drug side effects, and lowering drug development costs.

A promising platform for studying complex organ interactions and disease mechanisms are multi‐organ chips, including GBA chips. While these chips have shown the potential to provide a more physiologically relevant model for drug testing and disease modelling, there are still several technical challenges that need to be addressed for their further development. The incorporation of advanced microfluidic systems to better mimic the complex physiological environment of the gut and brain will be a key aspect of the technical analysis for the future development of gut–brain axis chips. In addition, for real‐time analysis of cellular and molecular responses within the gut–brain axis chips, the integration of advanced sensing and monitoring technologies will be critical. Furthermore, physiological relevance and predictive capabilities can be improved by incorporating organoids and stem cell‐derived tissues into the GBA chips. These advances will not only make it easier to study the interactions between the gut and the brain, but will also provide insights into complex diseases such as inflammatory bowel disease and neurodegenerative diseases. Overall, the future development of GBA chips will need to be multidisciplinary, combining microfluidic, tissue engineering, biosensor and manufacturing expertise to overcome technical barriers and realize the full potential of these innovative platforms.

## AUTHOR CONTRIBUTIONS

Li‐Guo Liang & Yu Zhang designed the study; Yu Zhang, Si‐Ming Lu and Jian‐Jian Zhuang writing‐original draft preparation and revised; Supervision, Li‐Guo Liang, Yu Zhang; Funding acquisition, Li‐Guo Liang, Si‐Ming Lu. All authors reviewed the manuscript and all authors have read and agreed to the published version of the manuscript.

## FUNDING INFORMATION

This study was supported by the Zhejiang Provincial Natural Science Foundation of China (LTGY23H160001), Zhejiang Provincial Health Major Science and Technology Program (WKJ‐ZJ‐2434) and Key Laboratory of Biomarkers and In Vitro Diagnosis Translation of Zhejiang province (KFJJ2023012).

## CONFLICT OF INTEREST STATEMENT

The authors have no conflicts of interest to declare that are relevant to the content of this article.

## Data Availability

All relevant data are within the article.
